# Nutrient Balancing by a Wild Browsing Herbivore: Nutritional Geometry of Snowshoe Hares (*Lepus americanus*)

**DOI:** 10.1002/ece3.72347

**Published:** 2025-10-18

**Authors:** Juliana Balluffi‐Fry, Lisa Shipley, Ruurd T. Zijlstra, Edward W. Bork, Murray Humphries, Stan Boutin

**Affiliations:** ^1^ Department of Biological Sciences University of Alberta Edmonton Alberta Canada; ^2^ School of Environment Washington State University Seattle Washington USA; ^3^ Department of Agricultural, Food, and Nutritional Sciences University of Alberta Edmonton Alberta Canada; ^4^ Department of Natural Resource Sciences McGill University Montreal Quebec Canada

**Keywords:** feeding trial, nutrition, nutritional geometry, snowshoe hares

## Abstract

Browsing herbivores must consider food digestibility while balancing the intake of multiple nutrients (i.e., protein and energy) simultaneously. Nutritional Geometry (NG) is a framework that is used to assess how nutrients interact to impact animal feeding behavior and body condition. Here, we use NG combined with detailed digestibility trials to evaluate how snowshoe hares ( *Lepus americanus* ), a common boreal browser that experiences 10‐year population cycles, balance energy and protein. We conducted 65 no‐choice and 15 multi‐choice feeding trials on 17 hares in Kluane, Yukon (Canada) during the winters of 2022 and 2023. We tested four diets ranging from the low protein (5.6%) and high fiber content of hare winter food (twigs) to the high protein (16.7%) and low fiber content of rabbit chow. We measured daily intake per kg^0.75^ per day in multi‐choice trials and daily intake, weight change, and digestibility in no‐choice trials. We analyzed the effect of diet treatment on each response and the effect of protein and energy intake, in both crude and digestible terms, on feeding rates and weight change. In multi‐choice trials, hares chose a diet balanced in energy and protein, but with a protein content above that in twigs. On single diets, hares were fed to meet a minimum daily digestible energy intake of approximately 1000 kJ/kg^0.75^/day, regardless of protein content, after which digestible protein influenced weight change (*p* = 0.02). We found that hares could maintain their weight after they acquired 6 g of digestible protein per kg^0.75^/day. Our results suggest that snowshoe hares choose to consume food items on the basis of the interaction between energy and protein, and these choices influence weight change. Our work supports previous hypotheses that declines in twig quality at peak hare densities could contribute to the subsequent increase in over‐winter weight loss that occurs during the population crash.

## Graphical abstract

Here, we use nutritional geometry and classical feeding trial methods to evaluate how snowshoe hares balance energy and protein. Our results suggest that snowshoe hares choose to consume food items on the basis of the interaction between energy and protein, and these choices influence weight change. Our work supports previous hypotheses that declines in twig quality at peak hare densities could contribute to the subsequent increase in over‐winter weight loss that occurs during the population crash.

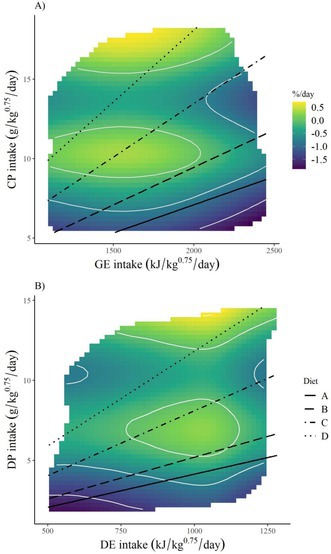

**Abbreviations**
ADF: acid detergent fiber
ADL: acid detergent lignin
CP: crude protein
DE: digestible energy
DM: dry matter
DMI: dry matter intake
DP: digestible protein
edf: effective degrees of freedom
GAM: general additive model
GE: gross energy
NDF: neutral detergent fiber
NG: nutritional geometry

## 1 Introduction

Animals must employ a balancing act of multiple food components simultaneously to maintain nutritional homeostasis, so it can be difficult to infer exact nutritional limitations (Demi et al. 2021; Felton et al. 2018; Santilli et al. 2023; Villalba and Provenza 2007). Prolonged nutrient (e.g., protein, energy, minerals) deficiencies can cause a decrease in reproductive output or survival (McArt et al. 2009; Simpson et al. 2010). Bertrand's rule states that surpluses of key nutrients can also reduce animal performance, usually defined as body condition or reproductive output, because of costs associated with removing excess nutrients (Bertand 1912; Raubenheimer et al. 2005). For herbivores, plant food often comes with structural (fiber) and chemical (secondary metabolites) compounds that decrease digestibility, another consideration on top of nutrient balancing (Felton et al. 2021). Plant digestibility can affect herbivores across many scales, from their foraging choices (Ellsworth et al. 2013) to their species distributions and functional traits (Lee 2018).

Nutritional geometry (NG) is a multi‐currency framework to graphically show how different food components or nutrients interact to affect behavioral and fitness outcomes (Simpson and Raubenheimer 2012). On the basis of Bertrand's rule, NG assumes that animals often balance the intake of multiple nutrients (Anderson et al. 2020; Felton et al. 2018). Using feeding experiments, NG measures animal behavior (e.g., daily intake) and performance in response to “nutritional space”, or two or more axes that represent nutrient intake (i.e., amount‐based) or composition (i.e., proportion‐based; Simpson and Raubenheimer 2012). First, studies use multi‐choice feeding trials, whereby individuals are offered multiple trial foods of varying nutritional ratios, to measure nutrient intake rates under ideal circumstances and identify a “target nutrient intake” (Senior et al. 2015). Next, a series of no‐choice trials measure the animal's daily intake and performance in response to individual foods (e.g., Krabbe et al. 2019). No‐choice feeding responses are plotted in nutritional space to understand how animals behave when restricted to one diet composition, like whether they meet a minimum intake of one nutrient or stay closest to the target intake (i.e., rules of compromise; Simpson and Raubenheimer 1995). From no‐choice trials, performance can also be plotted as a surface heat map in response to nutrient intake (e.g., Wilkinson et al. 2014). If an animal forages adaptively, the target intake from multi‐choice trials should fall within the area of nutritional space that yields the highest performance. With these methods, NG can uncover unique behavioral responses like nutrient balancing or trade‐offs among nutritional attributes. For example, studies have found trade‐offs between reproductive fitness and lifespan in flies when reared under different protein‐carbohydrate balances (Jang and Lee 2018).

Although NG has expanded from its original insect model systems, now being used to study humans (Wali et al. 2020), domestic animals (Hewson‐Hughes et al. 2013; Wilkinson et al. 2014), and wild vertebrates (Coogan et al. 2014; Guo et al. 2018; Rothman et al. 2011), it has been less often applied to browsing herbivores (but see Felton et al. 2016), possibly because of the difficulty of evaluating nutrient digestibility in highly fibrous and toxic foods. Digestibility trials that are based on the ratio of nutrient intakes (i.e., feeding rates) to outputs (i.e., total feces and urine) are the best way to measure food digestibility. NG studies on wild animals usually assess nutrient balancing or limitations by observing natural diets (e.g., Felton et al. 2009), but such studies cannot measure food digestibility (but see Felton et al. 2021; Hecker et al. 2021; Raubenheimer et al. 2015 for alternative strategies). For browsers, fitness and behavioral responses could change according to whether nutrient axes are represented in crude or digestible terms because the amount of nutrients a browser attains from a food often does not equate to the amount of nutrients used by the animal for performance.

Here, we use wild‐caught snowshoe hares ( *Lepus americanus* ) held temporarily in captivity in winter as a case study to experimentally apply NG to wild browsers using digestive feeding trials. Snowshoe hares are a small (~1.3 kg), monogastric herbivore that exist across the North American boreal forest and exhibit 10‐year population cycles (Krebs et al. 2018). They are income breeders with high metabolisms and low body fat, making them susceptible to negative energy and nutrient balances (Whittaker and Thomas 1982). Hares are most food‐limited during winter, when available plant biomass is predominantly dormant twigs on shrubs (Smith et al. 1988). Because shrubs become more lignified during dormancy, twigs in winter are lower in protein (~5% Rodgers and Sinclair 1997), more fibrous, and less digestible than twigs in spring and summer (~20% protein; Seccombe‐Hett and Turkington 2008). Snowshoe hares tend to lose body weight over winter (Hodges et al. 2006), and studies have found that heavier females in late winter have a greater total mass of first litters that spring (Majchrzak et al. 2022). Thus, the rate of weight loss is often used as an index for body condition in hares (Hodges et al. 2006; Rodgers and Sinclair 1997). Controlled studies found that food supplementation reduced weight loss and female reproductive output (Hodges et al. 2006). However, the supplement in these studies consisted of a pelleted, grain‐alfalfa rabbit chow, which is much higher in energy and protein than twigs; thus, it was unrepresentative of the foraging conditions experienced by wild hares during winter (e.g., Majchrzak et al. 2022; Smith et al. 1988). The nutritional implications of high protein food treatments on hare feeding behaviors have not been thoroughly investigated, but there is some evidence that browsers balance unnaturally high protein and energy diets with fiber (Felton et al. 2017; Hodges and Sinclair 2003).

Older studies on hare nutrition have found mixed results regarding nutrient limitation. Sinclair et al. (1982) suggested that hares are protein‐limited overall, but Pehrson (1984) rebutted, suggesting that hares are more energy‐limited on the basis of feeding trials using mountain hares ( *Lepus timidus* ; Pehrson 1983). Later, Rodgers and Sinclair (1997) concluded that hare nutrition involves the interaction between protein, energy, and plant secondary metabolites. Because NG specifically evaluates how multiple nutrients interact to affect animal behavior and performance, it could help shed light on the conflicting results of previous studies.

In this study, we addressed two objectives: (1) to apply the NG framework to controlled feeding trials to better understand hare nutritional limitations and (2) to determine the degree to which geometric responses by a browsing herbivore differ when using crude protein (CP [%]; estimated from measurements of nitrogen) and gross energy (GE [kJ/g]; measured from heat given off when a food sample is completely combusted) compared to more biologically relevant measures that account for slow and indigestible portions of plant diets. The latter would reflect the amount of protein and energy the animal can digest, or the digestible protein (DP) and digestible energy (DE), respectively. For the first goal, we sought to identify how limits and surpluses in protein and energy influenced daily intake and weight loss. We used a breadth of food compositions, ranging from low‐quality twigs to rabbit chow. To do so, we used NG to identify the snowshoe hare target intake, rules of compromise, and performance in response to crude and digestible forms of energy and protein. In accordance with Rodgers and Sinclair (1997), we predicted that hares will respond best to a balanced intake of protein and energy, meaning that the target intake and area of highest performance will be in the middle of our available nutritional space, and that hares will feed to be closest to that target intake. We also predicted that DE intake would explain more variation in performance than GE intake.

## 2 Methods

### 2.1 Study Area

We conducted feeding experiments with snowshoe hares at the Kluane Lake Research Station of the Arctic Institute of North America in the Kluane region of the southwest Yukon, Canada (coordinates) during February and March of 2022 and 2023. All handling and collaring procedures were approved by the University of Alberta Animal Care Committee (AUP00001973) and conducted in accordance with research permits issued by the Government of Yukon. The Kluane Valley is the site of a long‐term research program that has monitored and studied the snowshoe hare population cycle for over 40 years using established live trapping methods and grids (Krebs et al. 2018). The long‐term monitoring aspect of this project involved mark‐recapture to estimate hare densities each September, after the reproductive season, and each April, after some of the population is lost to winter predation. This project took place during the low and early increase phase of the snowshoe hare cycle (Krebs et al. 2023). The spring hare densities during this study were estimated to be 0.058 and 0.15 hares/ha in 2022 (Krebs et al. 2023) and 2023 (unpublished), respectively. The vegetative cover is predominantly white spruce ( *Picea glauca* [Moechin]) with some aspen stands ( *Populus tremuloides* [Michx.]). Willow (*Salix* spp. [Host]), bog birch ( *Betula glandulosa* [Michx.]), and Canada buffaloberry ( *Shepherdia canadensis* [Nutt.]) shrubs dominate the understory. The most available and twig species for snowshoe hares in the area are spruce and willow (Rodgers and Sinclair 1997). Previous research in Kluane has used both free‐range and individual‐based food supplementation experiments to test the effects of food on snowshoe hare body condition, survival, and reproduction (Sinclair et al. 1988; Majchrzak et al. 2022).

### 2.2 Experimental Diets

**FIGURE 1:**
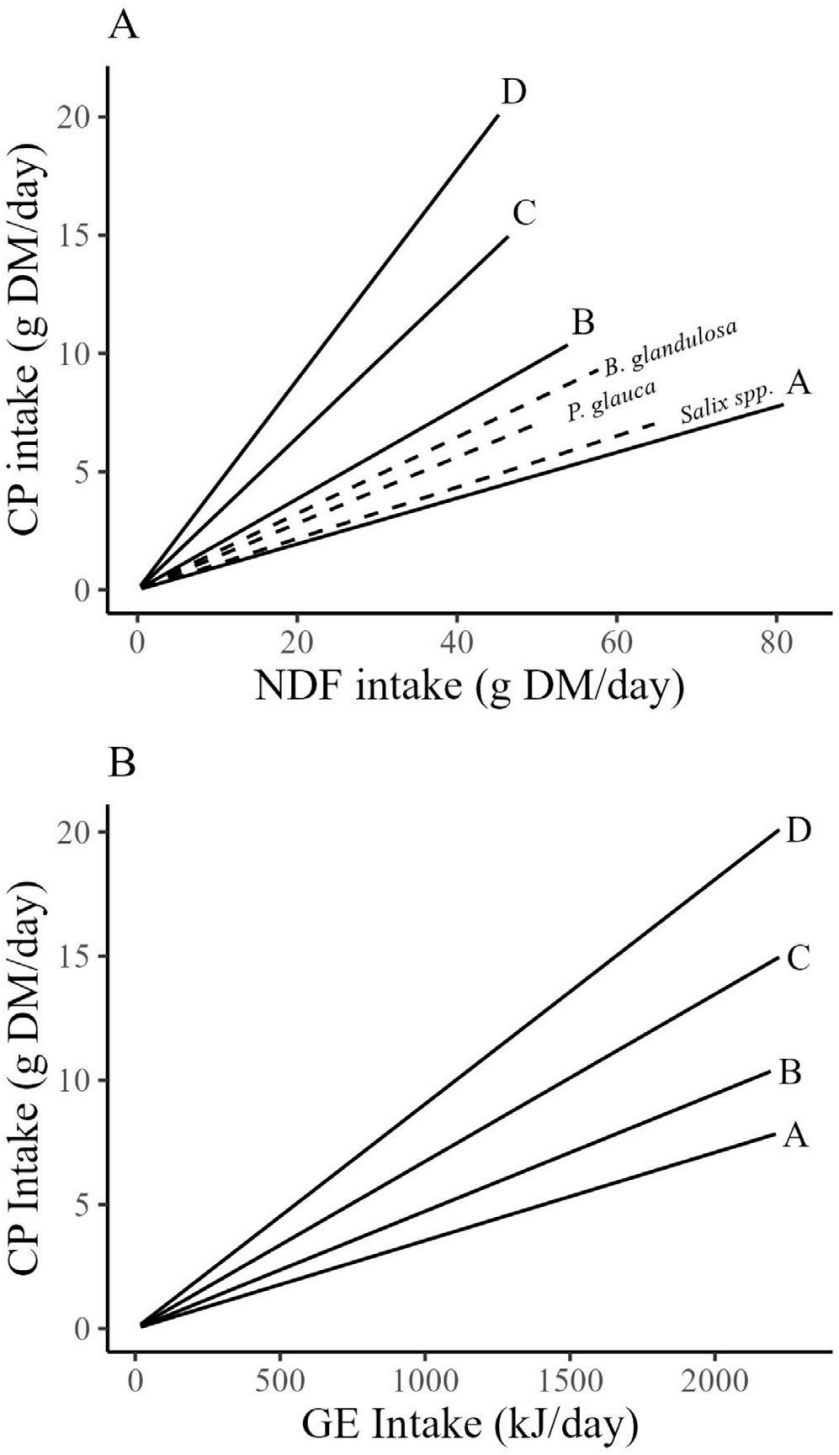
Design of formulated diets for snowshoe hare ( *Lepus americanus* ) feeding trials, conducted in Kluane, Yukon, in February and March of 2022 and 2023, in terms of (A) crude protein (CP) and neutral detergent fiber (NDF) and (B) CP and gross energy (GE). Diets are shown as nutritional rails, which represent the intake of each respective food component if the hare were to feed solely on that diet. Diets A, B, C, and D were designed to have CP:NDF ratios of 0.083, 0.22, 0.36, and 0.5, respectively. For comparison, we show CP:NDF of three common winter browse species, *Betula glandulosa* , *Picea glauca* , *and Salix* spp. (dashed lines), which we measured from samples collected in the study area. The subsequent CP:GE ratios measured post‐pelleting are shown in panel B.

By manipulating the proportion of 6 ingredients that differed in CP and neutral detergent fiber (NDF) content, we created four diets that ranged from emulating the low protein and low energy content typical of twigs during winter (Rodgers and Sinclair 1997) to the high protein and high energy content of rabbit chows used in previous food supplementation studies (e.g., Majchrzak et al. 2022). We milled and pelleted diets at the University of Saskatchewan Canadian Feed Research Centre (North Battleford, SK, Canada). The lowest protein diet, Diet A, was designed to have 5.3% CP and a CP:NDF ratio of 0.083, whereas the highest protein diet, Diet D, would have 15% CP and a CP:NDF of 0.50 (Figure 1A and Table 1). Diets ultimately had similar GE content (Table 1). These formulations resulted in the desired progressive increase in CP:GE ratio from Diet A to Diet D (Figure 1B). Diet A and Diet B represent the lower and upper limits of protein content in natural twigs, respectively (Figure 1). Diet D represented rabbit chow that is high in protein and energy. We analyzed the composition of all diets post‐pelleting, and actual diet compositions varied slightly from their original calculations (see Table 1 for actual compositions).

**TABLE 1:** Ingredient and nutrient compositions of pelleted experimental diets fed to snowshoe hares ( *Lepus americanus* ) in feeding trials conducted in Kluane, Yukon, in February and March of 2022 and 2023.

Item	Diet (CP:NDF)			
	A (0.083)	B (0.22)	C (0.36)	D (0.50)
*Ingredient (%)*				
Oat hulls	54.6	29.4	26.5	30.3
Cellulose	8.6	2	0.39	—
Timothy hay	26.7	28.7	25.8	17.3
Soybean meal	—	7.4	15.3	23.8
Corn starch	6.11	28.5	27.9	24.5
Molasses	4	4	4	4
Limestone	0.3	—	—	0.08
Salt (Sodium chloride)	0.01	0.03	0.03	0.02
*Analyzed nutrient content, dry matter basis*				
Crude protein (CP, %)	6.5	8.6	12.5	16.7
Neutral detergent fiber (NDF, %)	67.4	44.9	38.7	37.7
Acid detergent fiber (ADF, %)	40.0	26.1	22.2	21.5
Acid detergent lignin (ADL, %)	5.0	3.6	3.2	3.0
Gross energy (GE, kJ/g)	18.4	18.3	18.5	18.5
*Apparent digestibility, dry matter basis*				
Dry matter (%)	36.3	55.2	57.7	59.2
Gross energy (kJ/g)	6.7	10.1	10.7	11.0
Crude protein (%)	2.8	5.2	8.7	13.0

*Note:* Diets were formulated (ingredient composition) to have linearly increasing crude protein to neutral detergent fiber ratios from 0.083 (Diet A) to 0.5 (Diet D) while maintaining a constant neutral detergent fiber to acid detergent fiber ratio of 0.6. Apparent digestibility values were estimated in vivo from no‐choice feeding trials.

We ensured that diets had similar fiber compositions. Of the total NDF, 57%–59% was from acid detergent fiber (ADF) and 7%–8% was from acid detergent lignin (ADL). Diets had NDF:ADF ratios similar to those of dormant twigs we analyzed (twig samples used for Figure 1A). We also balanced all diets with salt (sodium chloride) and limestone to control for sodium and calcium content (Table 1; Worker et al. 2015; Kaspari 2020). We ground all ingredients through a 3.6‐mm screen in a hammer mill and steam‐pelleted the mixture at 70°C. Pellets were 4.5 mm in diameter and ~130 mm in length.

### 2.3 Experimental Enclosures

We temporarily housed hares in enclosures inside an unheated building with large windows that subjected them to natural winter temperatures and light. Eight enclosures (0.5 m wide, 1 m deep, and 1 m tall) were made of wire mesh and plywood. The enclosures were elevated and had mesh wire floors (1 × 1 cm) to let fecal pellets and urine pass. Below each enclosure was a finer mesh to catch fecal pellets. This experimental design did not interfere with coprophagy (i.e., re‐ingesting feces or cecotropes as part of digestion) because lagomorphs consume cecotropes directly from their anus (Santilli et al. 2023). Inside each enclosure, we secured a small shelter and a bin to hold snow as a water source. Snow was replenished daily.

### 2.4 Experimental Procedure

In February and March of 2022 and 2023, we conducted 65 no‐choice feeding trials and 15 multi‐choice feeding experiments on 17 snowshoe hares (F = 7, M = 10, average weight = 1293 ± 68.4 g). To collect animals, we trapped the snowshoe hares using Tomahawk live traps (Tomahawk Live Trap Co., Tomahawk, Wisconsin, USA) within 25 km of the research station. Upon capture, we ear‐tagged hares and assessed their weights with a Pesola scale to the nearest 20 g. Total captures were 17 and 16 individuals for 2022 and 2023, respectively. We only used hares for feeding trials if they weighed more than 1100 g. We transported eligible hares back to the research station in separate, secured burlap sacs. When at the research station, we weighed hares more precisely on a kitchen scale (nearest 5 g) before transferring them into separate enclosures (Figure S1). No mortalities were caused by trapping, handling, or experimentation.

Hares are notoriously difficult to keep in captivity because they stress easily; therefore, our experiments were designed to minimize the total length of time hares would be in captivity. The first two days of the experiment served as the habituation period and multi‐choice feeding trial (Figure S1). On the first day, we conducted the naïve multi‐choice feeding experiment to measure preference among diets and the target nutrient intake by providing hares with ~75 g of all four experimental diets. After 24 h, we weighed the remaining food (g) to calculate the total amount consumed and sampled 10 g of each diet to later assess dry matter (DM) content. For the next day, we further habituated hares to diets and enclosures by providing them all four experimental diets, commercial rabbit chow, a mixture of twigs (*Salix* spp., *P. glauca* , and *P. balsamifera* ), apple, and alfalfa ( *Medicago sativa* ) cubes. We weighed hares again following these two nights of habituation. Hares always ate formulated diets by the end of the two‐day habituation phase.

After habituation, hares participated in four no‐choice feeding trials, one per diet, to measure their daily intake (g DM/kg^0.75^/day), weight change (%/day), and apparent digestibility of dry matter, energy, and protein (%) for each diet separately (Figure S1). We randomized the order of diets for each hare. Feeding trials lasted three consecutive days in which we offered a hare one diet and snow, ad libitum. Daily, we weighed the offered food (g) and remaining food (g), took a 10 g sample of diets to later measure DM, and collected all fecal pellets produced. We weighed hares (g) at the start and end of the feeding trial and calculated the resulting weight change as a percentage of their starting weight using the following calculation:

$$\text{Weight change}\;\left(\%\mathrm{per}\;\mathrm{day}\right)=\frac{\mathrm{end}\;\text{weight}-\text{start weight}}{\text{start weight}\times 3}\times 100$$

We kept all diet and fecal samples frozen at −20 C until further analysis. Feeding trials were separated by 2‐day recovery periods (Figure S1). After feeding trials, hares were again offered all diets: commercial rabbit chow, apple, and alfalfa cubes to recover any weight lost during the prior feeding trial. After recovery, hares would begin the subsequent feeding trial, and this was repeated until all four sequential trials were complete, after which hares were released at their original trap location (Figure S1).

We required hares to meet certain weight criteria after each recovery period to continue in the experiment. Snowshoe hares can occasionally become stressed during captivity to the point of “food strike” and lose weight as a result (Rodgers and Sinclair 1997). If a hare lost more than 5% of its initial weight during habituation, it was released and not used in further trials (*n* = 2), and data were discarded. During no‐choice trials, some hares did not recover weight during recovery periods and instead continuously lost weight during the experiment. We pre‐emptively released any hare that reached a net weight loss of 8% from their initial weight at first capture after a recovery period (*n* = 4). We released these individuals at their original trap location and re‐trapped them (identified by ear tag) at least 7 days later to continue feeding trials. All hares had regained weight (< 5% change from initial weight) by the time they were re‐trapped. We used results from hares that were released and re‐trapped. Thirteen hares successfully completed the entire feeding trial course without interruption. Hares are nocturnal feeders and have short (< 12 h) gut passage times. In the few cases where a hare refused to eat overnight, it did not produce any feces.

### 2.5 Chemical Analyses and Calculations

To estimate daily intake and apparent digestibility of each diet in terms of DM, energy, and protein, we measured DM (%) and CP (%) content of diets and feces; to limit costs, we only measured the GE of diets and not fecal samples. We measured GE (kJ/g) of diets using a bomb calorimeter (IKA C 200 Calorimeter). Nitrogen concentration was determined using a flash auto‐analyzer (ThermoFisher Flash 2000), which was then multiplied by 6.25 to estimate CP content. To confirm diet NDF, ADF, and ADL (Table 1), we used a sequential analysis of samples placed in mesh bags and a fiber analyzer (Ankom 200 Fiber Analyzer, Macedon, NY, USA). Next, we analyzed all feed and fecal samples for DM content by weighing subsamples before and after drying at 100°C for 24 h (DM = wet weight/dry weight × 100).

All food intake, fecal output, and diet digestibility were estimated for each feeding trial day. We corrected the diet and feces for DM content. We calculated dry matter intake (DMI; g/day) as the difference between the dry mass of remaining and offered food. Next, we calculated CP (g DM/day) and GE (kJ/day) intake and output as the product of DMI × diet CP and GE, respectively. We estimated the CP output (g DM/day) as the product of DM output × fecal CP. Intakes and outputs were used to calculate the apparent digestibility of CP and DM:

$$\text{apparent digestibiltiy}\;\left(\%\right)=\left(\frac{\mathrm{DM}\;\text{input}-\mathrm{DM}\;\text{output}}{\mathrm{DM}\;\text{input}}\right)\times 100$$

where ‘input’ (i.e., eaten) and ‘output’ (i.e., excreted) represent any macronutrient, food component, or the whole food item (Tassone et al. 2020). Given our inability to analyze each fecal sample for GE, we estimated energy digestibility (kJ/g) as the product of total dry matter digestibility/100 × diet GE. We estimated digestible protein (DP) intake (g DM/day) as the product of CP intake × diet protein digestibility/100. We estimated digestible energy (DE) intake (kJ/day) as the product of DMI × diet energy digestibility. All daily intakes were converted to a per kg^0.75^ basis using the starting body weight of the trial.

### 2.6 Replication Statement

**Table:** 

Scale of inference	Scale at which the factor of interest is applied	Number of replicates at the appropriate scale
Individuals‐trial	Diet treatment	17 individuals, 4 diets per individual, 1 trial per diet
Individuals‐day	Diet treatment	17 individuals, 4 diets per individual, 3 days per diet

### 2.7 Statistical Analyses

We first evaluated the effects of diet (i.e., treatment) on snowshoe hare DMI, weight change, dry matter digestibility, and protein digestibility using analyses of variance, with diet as the only fixed effect. We ran a post hoc mean comparison using a Tukey test to determine significant differences between each combination of diets (*p* < 0.05). First, using results from naïve multi‐choice trials, we tested whether DMI differed by diet to determine diet preference. Next, with results from the no‐choice feeding trials, we tested if hare weight change, DMI, dry matter digestibility, and protein digestibility differed by diet. Weight change responses were measured on a trial basis (i.e., at the end of each 3‐day feeding trial), whereas feeding and digestibility responses were measured daily (i.e., three measures per trial). Standard errors (±) are reported in the text.

To assess feeding behavior in terms of nutritional space, we plotted feeding responses in terms of both crude and digestible daily intake. We calculated the total GE, CP, DE, and DP ingested by hares in multi‐choice trials and used the average of each as target intake rates. Next, we plotted the average DMIs of diets in no‐choice trials on their respective nutritional rails, in terms of GE:CP and DE:DP, to assess how hares respond behaviorally to surpluses and deficits of each nutrient.

Next, from the no‐choice trial results, we assessed patterns of weight change in response to daily DMI of CP, GE, DP, and DE, our form of nutritional space. We ran two generalized additive models (GAMs), one for CP versus GE and one for DP versus DE. GAMs are a type of generalized linear model that allows terms to be non‐parametric or “smooth” to test whether relationships are non‐linear. Both models included the interaction term between the two respective daily intakes. GAMs were fit using the ‘gam’ function in the *mgcv* package (Wood 2023). We evaluated the linearity of each model using the effective degrees of freedom (edf), with an edf of 1 being a linear relationship and an edf > 2 being a non‐linear relationship. The higher the edf, the more non‐linear the relationship. To visualize how weight change responded to this nutritional space, we used the ‘predict_gam’ function to use GAM outputs to predict weight change across both types of nutritional space. We plotted the raster and contour plots of these predictions using the ggplot2 package, with energy intake on the *x*‐axis and protein intake on the *y*‐axis (Wickham 2016).

Lastly, to compare with non‐NG studies, we used results from no‐choice trials to linearly estimate CP, GE, DP, and DE intake requirements for hares (i.e., the minimum intake rate required for body maintenance or zero weight loss). We ran four linear models that tested weight change in response to CP, GE, DP, and DE separately. If a relationship between intake rate and weight change was present (*p* < 0.05), we then extracted the intake rate where the linear regression intercepted the zero line (i.e., the value at which the hare weight change was zero).

## 3 Results

### 3.1 Multi‐Choice Trials

**FIGURE 2:**
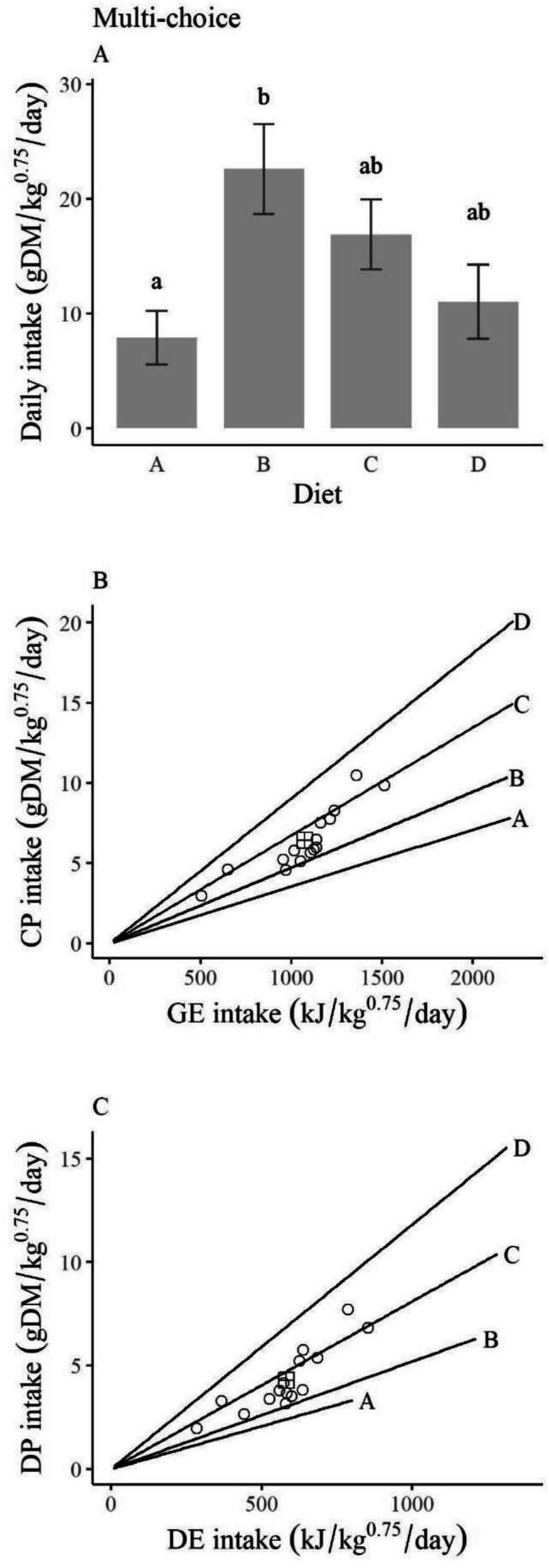
Feeding responses by snowshoe hares ( *Lepus americanus* ) to diets in multi‐choice feeding trials (*n* = 15) conducted in Kluane, Yukon, in February and March of 2022 and 2023. (A) Median daily intakes of each diet (g DM/kg^0.75^/day; error bars = standard error) where different letters denote significant differences among means (*p* < 0.05). (B) Daily intake in terms of crude protein (CP; g DM/kg^0.75^/day) and gross energy (GE; kJ/kg^0.75^/day). (C) Daily intake in terms of digestible protein (CP; g DM/kg^0.75^/day) and digestible energy (DE; kJ/kg^0.75^/day). In B and C, lines represent the ratios of the nutrients within each diet, circular points represent the total nutrient intakes by individuals in trials, summing their intake of all diets, and the square cross point represents the mean nutrient intakes of all individuals, that is, a potential target intake.

In multi‐choice trials, snowshoe hares ate an average of 58.4 ± 5.1 g DM/kg^0.75^/day across all diets. There was a significant effect of diet on DMI (*p* = 0.01). DMI of Diet B was 2.9 times that of Diet A (*p* = 0.01) and 2 times that of Diet D (*p* = 0.06; Figure 2A). DMIs across all diets during the multi‐choice experiment translated to an average daily intake of 6.4 ± 0.8 g DM/kg^0.75^/day of CP and 1074.7 ± 94.4 kJ/kg^0.75^/day of GE (Figure 2B). Average daily intake of DP was 4.3 ± 0.6 g DM/kg^0.75^/day and DE was 581.8 ± 54.6 kJ/kg^0.75^/day of DE (Figure 2C). When plotted in nutritional space, the target intakes of CP and GE and DP and DP fell between the nutritional rails of Diets B and C (Figure 2B,C).

### 3.2 No‐Choice Trials: Feeding Responses

**FIGURE 3:**
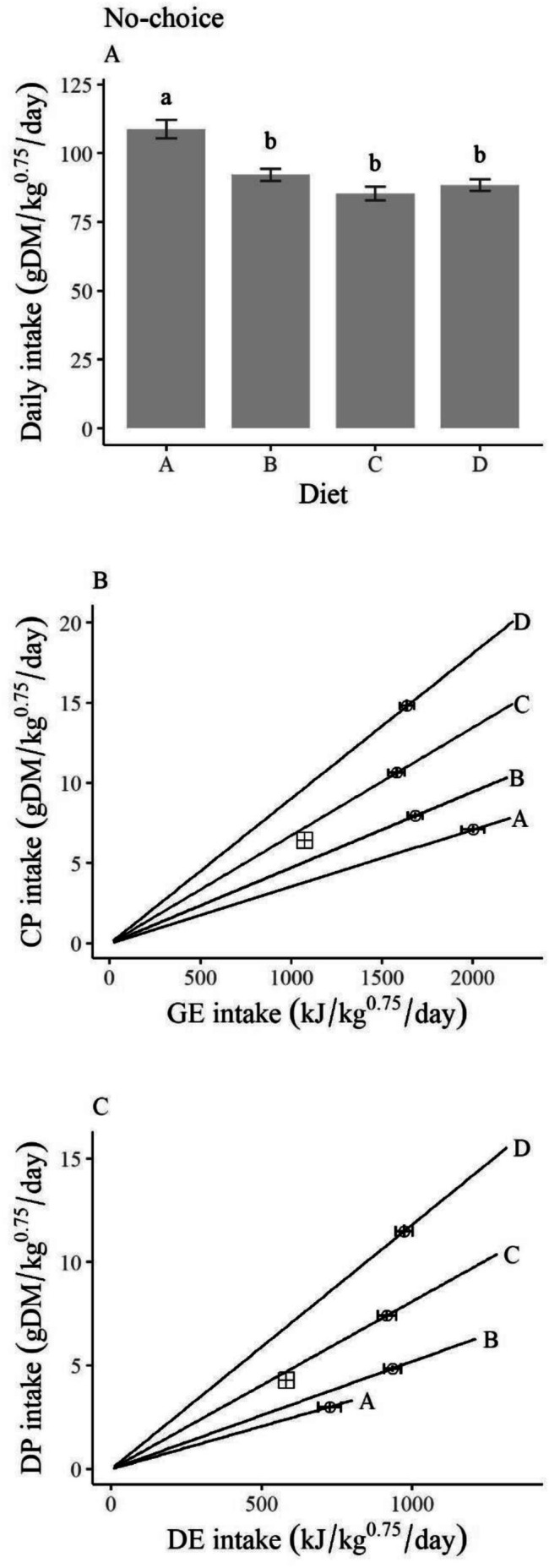
Feeding responses by snowshoe hares ( *Lepus americanus* ) to diets in no‐choice feeding trials (*n* = 65) conducted in Kluane, Yukon, in February and March of 2022 and 2023. (A) Median daily intakes of each diet (g DM/kg0.75/day; error bars = standard error) where different letters denote significant differences among means (*p* < 0.05). (B) Daily intake in terms of crude protein (CP; g DM/kg0.75/day) and gross energy (GE; kJ/kg^0.75^/day). (C) Daily intake in terms of digestible protein (CP; g DM/kg0.75/day) and digestible energy (DE; kJ/kg^0.75^/day). In B and C, lines represent the ratios of the nutrients within each diet, circular points represent the mean intake rates of each diet (error bars = standard error) in no‐choice trials, and the square cross point represents the mean nutrient intakes of all individuals, that is, a potential target intake.

Hares ate an average of 93.6 ± 3.2 g DM/kg^0.75^/day of feed across all diets. DMI for Diet A was significantly higher than all other diets (*p* < 0.001), whereas there was no difference between diets B, C, and D (Figure 3A). Hares ate about 25% more of diet A than the other diets (Figure 3A). In terms of CP and GE, no‐choice daily intake curved away from the target intake, appearing as though, on diets A and B, hares aim to meet a minimum CP intake while aiming to meet a minimum GE intake on diets C and D (Figure 3B). Using more biologically relevant nutritional metrics, DP and DE showed that hares appeared to meet a certain intake of DE (~900 kJ/kg^0.75^/day) regardless of DP (Figure 3C). On Diet A, hares could not achieve the DE intake of the other diets nor the target intake of DP that was observed in multi‐choice trials (Figure 3C).

### 3.3 No‐Choice Trials: Weight Change and Digestive Responses

**FIGURE 4:**
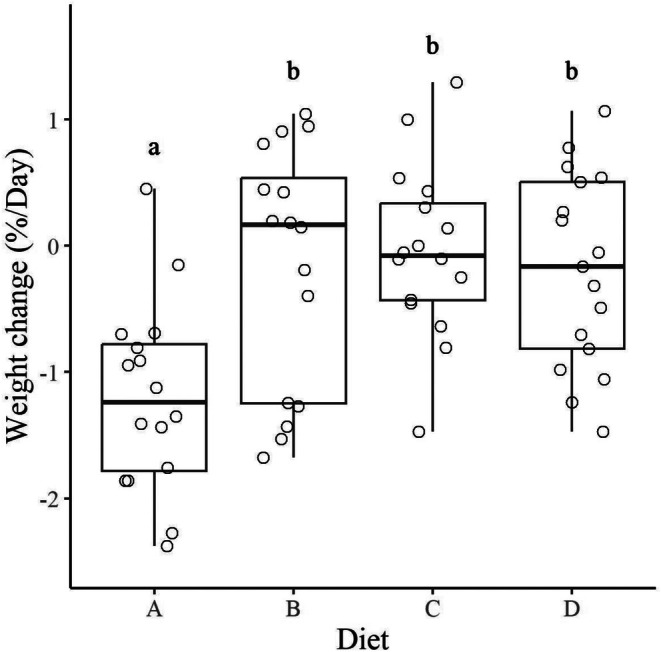
Weight change (% change from initial body weight/day) of snowshoe hares ( *Lepus americanus* ) in response to diet during three‐day, no‐choice feeding trials (*n* = 65) conducted in Kluane, Yukon, in February and March of 2022 and 2023. Boxes represent median weight change bounded by lower 25th and 75th percentiles. Circular points represent the values of individual feeding trials. Different letters denote significant differences among means (*p* < 0.05).

Diet affected changes in hare weight between the beginning and end of each 3‐day feeding trial (*p* < 0.001; Figure 4). Hares lost the most weight on Diet A (median = −1.2% per day). On average, hares could maintain weight on the other diets (Figure 4).

**FIGURE 5:**
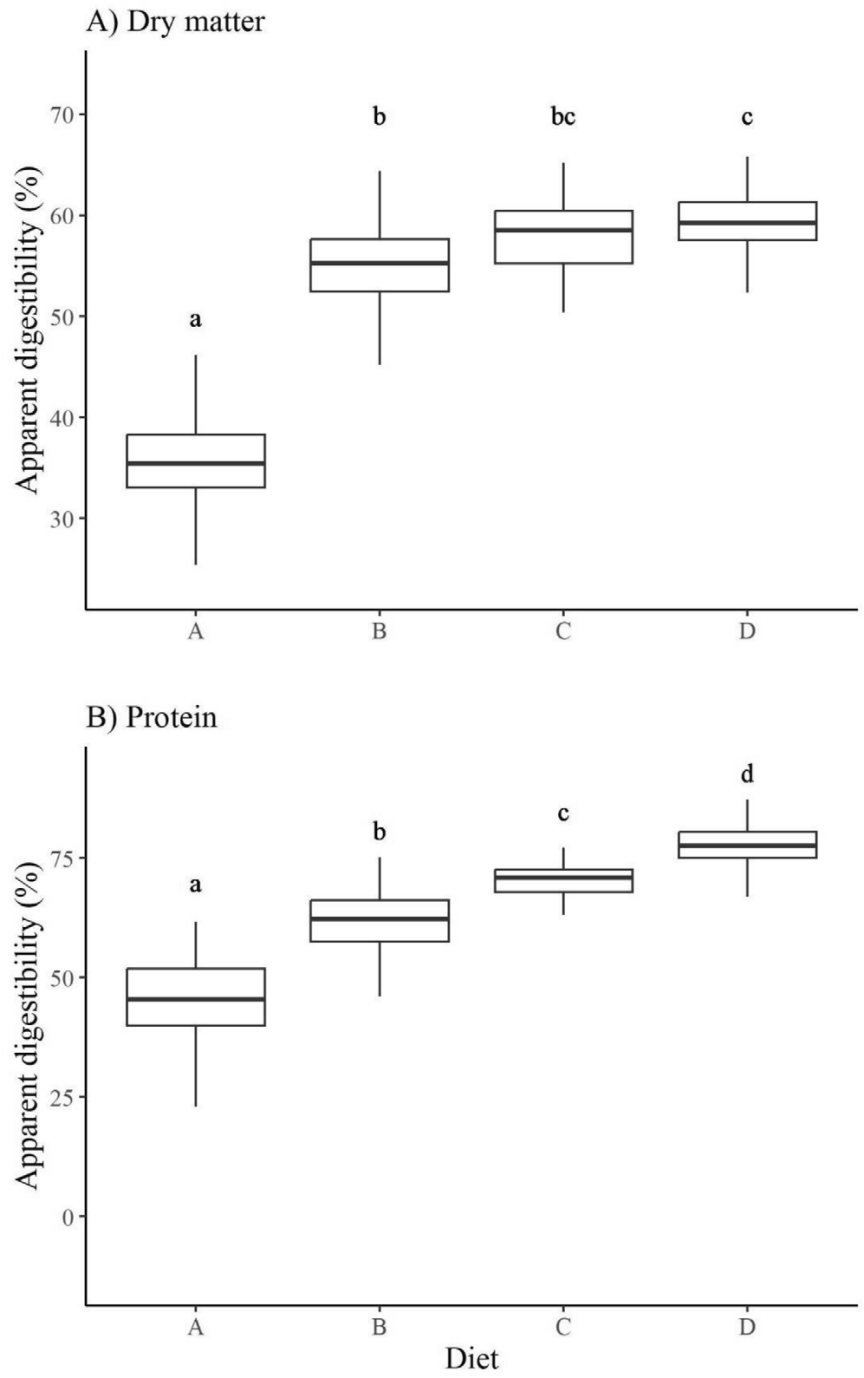
Daily apparent (A) dry matter and (B) crude protein digestibility (%) of snowshoe hares ( *Lepus americanus* ) in response to diet (*n* = 195; 65 trials) during no‐choice feeding trials conducted in Kluane, Yukon, in February and March of 2022 and 2023. Feeding trials measured digestibility daily (three per trial). Boxes represent median digestibility bounded by the lower 25th and 75th percentiles. Different letters denote significant differences among means (*p* < 0.05).

Like daily intake and weight change, dry matter digestibility differed across diets (*p* < 0.001; Figure 5A). Diet D was the most digestible (59.2%), and Diet A (35.6%) was the least. Diet A was 22% less digestible than other diets (*p* < 0.001), and Diet B was 3.9% less digestible than Diet D (*p* < 0.01; Figure 5A). CP digestibility increased from Diet A to D (*p* < 0.001; Figure 5B). Protein in Diet A was 41.9% digestible, whereas protein in Diet D was 77.3% digestible, 1.85 times greater than that of Diet A.

**FIGURE 6:**
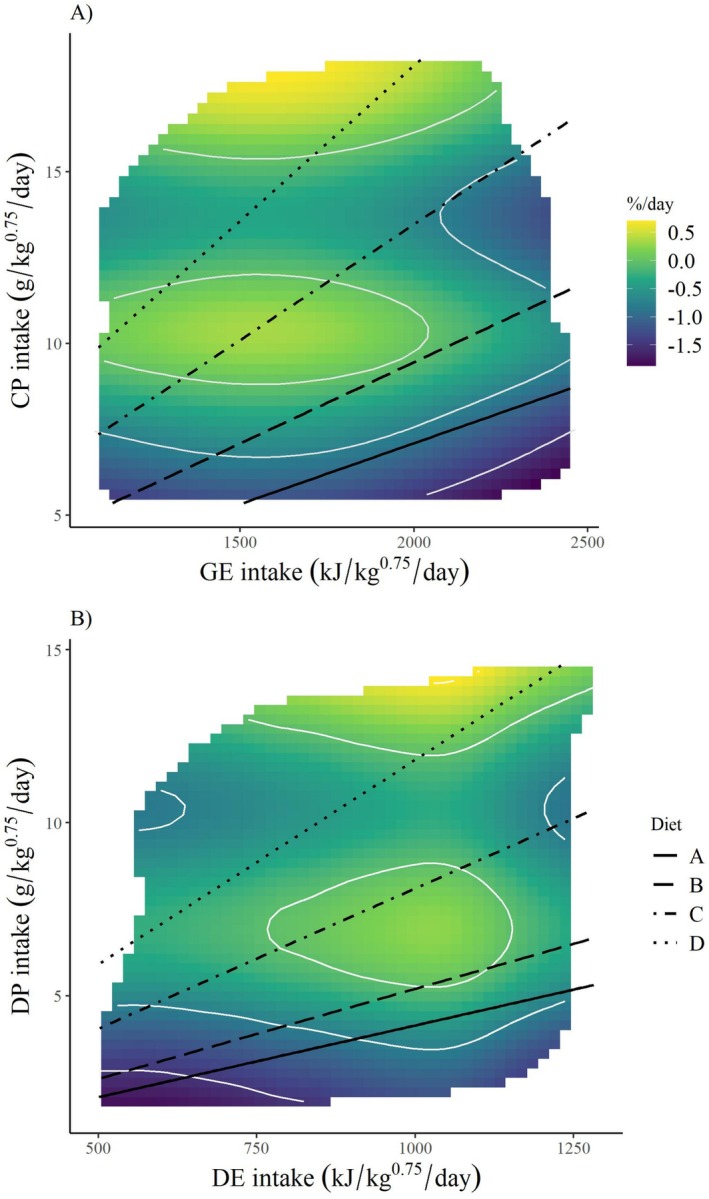
Daily weight change (%/day) of snowshoe hares ( *Lepus americanus* ) in response to dry matter intake rates of (A) crude protein (CP; g DM/kg^0.75^/day) and gross energy (GE; kJ/kg^0.75^/day) and (B) digestible protein (DP; g DM/kg^0.75^/day) and digestible energy (DE; kJ/kg^0.75^/day). Data was collected via feeding trials in Kluane, Yukon, in February and March of 2022 and 2023. Weight change predictions are shown as surface heatmaps with blue representing the greatest weight loss (negative value) and yellow representing weight gain (positive value). Predictions for weight change are derived from general additive models (Table 2) with low‐confidence values (standard error < 4) removed.

In terms of daily intake of GE (*x*‐axis) and CP (*y*‐axis) hares generally were able to maintain their weight when CP intake ≥ 9 g DM/kg^0.75^/day, but only when coincident with low and mid ranges of GE intake, such as occurred between the rails of diets B, C, and D. After GE intake surpassed ~2000 kJ/kg^0.75^/day, weight loss occurred regardless of protein intake (Figure 6A). The GAM associated with this surface map showed CP intake to have a significant, non‐linear effect on weight change and GE to have no effect (*R* ^2^ = 0.3; deviation explained = 0.37; Table 2). The surface map for DE and DP intake revealed one area in the center of the plot, between Diets B and C, where hares were able to maintain their weight, and this also occurred at the highest levels of DP intake, as exemplified by the highest intakes on Diet D (Figure 6B). The GAM for this map showed DP intake had a significant, non‐linear effect on hare weight change, whereas DE had no effect (*R* ^2^ = 0.35; deviation explained = 0.42; Table 2).

**TABLE 2:** Generalized additive model outputs for surface heat maps (Figure 5) that show results from two interactive models that predict weight change of snowshoe hares ( *Lepus americanus* ) from daily intake of either gross energy (GE) and crude protein (CP), or digestible energy (DE) and digestible protein (DP) food components (Model column).

Model	Intake term	edf	*F*	*p*
Crude	GE	1.97	2.58	0.07
	**CP**	**4.97**	**3.96**	**0.002**
	GE × CP	0	0	0.3
Digestible	DE	1.17	0.24	0.78
	**DP**	**4.01**	**2.98**	**0.02**
	DE × DP	1.89	0.1	0.13

*Note:* Results are from no‐choice feeding trials (*n* = 15) conducted in Kluane, Yukon, in February and March of 2022 and 2023. Smoothed term GAM model outputs include the nutrient intake term, effective degrees of freedom (edf), *F*‐values, and *p*‐values. Significant effects are bolded.

On the basis of linear regressions, our feeding trials estimated that hares require 1100 kJ/kg^0.75^ of DE, 14 g DM/kg^0.75^ of CP, or 12 g DM/kg^0.75^ of DP per day to maintain body weight (Figure 6). The relationship between GE intake and weight change was non‐significant (*p* = 0.48).

## 4 Discussion

Our study, which combined traditional nutritional techniques with a multi‐currency nutritional geometry (NG) model, has offered a deeper understanding of hare feeding behavior and nutrition. Our study supports what previous studies inferred—that food quality, via an interaction of nutrients, could play a key role in regulating snowshoe hare survival and potentially their population cycle (Rodgers and Sinclair 1997). Specifically, we found that hares feed to meet a minimum DE intake, after which protein intake affects weight change. We also found a narrower range of optimal nutrition using models that measured the intake of digestible nutrients (DE and DP) than when using cruder measures, GE and CP.

Our results suggest that many species of dormant twigs do not provide enough DP and/or DE for snowshoe hares to maintain body weight. Diet A, which represented the lowest quality twigs, caused the greatest weight loss, lowest digestibility, and highest daily intake (Figure 4). Hares were able to maintain their weight on Diets B, which represented the highest quality twigs, Diet C, and Diet D, despite eating less of these diets (Figure 4). In three trials, hares experienced negative protein digestibility from Diet A. Negative protein digestibility can occur when protein intake does not meet the basic animal requirements for fixed metabolic processes (Phillipson 1971). Like what Pehrson (1983) reported, we found that the higher the concentration of CP in a food, the higher the proportion of CP that was digested (Figure 5). In multi‐choice trials, hares chose a ratio of 7.4 g of DP per 1000 kJ of DE per kg^0.75^ per day, which is a higher protein: energy ratio than that typically found within twigs but lower than the highest protein contents available. One area of weight maintenance in heat maps fell around the protein: energy ratio of the target intake, suggesting that a diet balanced in energy and protein is optimal for hares.

We found evidence that energy and protein interact to affect total hare nutrition. Previous studies like Rodgers and Sinclair (1997) and Sinclair et al. (1982) inferred, but could not concretely visualize, this notion of nutritional interaction. Our study found that DMI of hares in no‐choice trials appears to be influenced by DE (Figure 3C), after which protein intake strongly affected performance. GAMs, which assessed how protein and energy interact to affect hare's performance, found that CP and DP predicted more of hare's weight change than DE and GE (Table 2). Future work that tests protein: energy ratios between Diets A and B could determine whether hares respond linearly or non‐linearly to this nutritional gradient. If hares respond non‐linearly, there may be a nutritional threshold supporting body maintenance somewhere between the composition of diet A and B.

**FIGURE 7:**
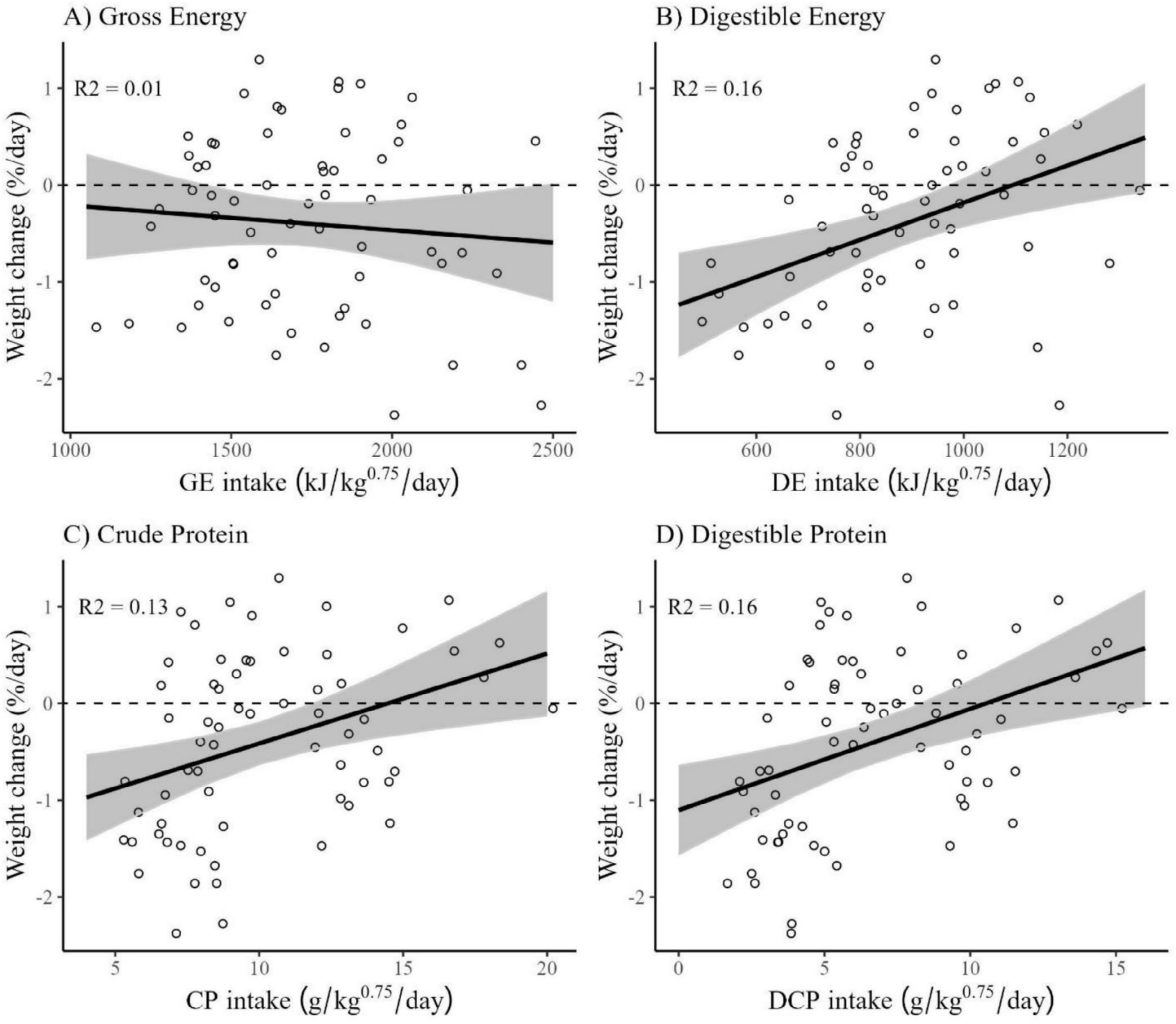
Linear response of weight change of snowshoe hares ( *Lepus americanus* ) to the dry matter intake of (A) gross energy (GE; kJ/kg^0.75^/day), (B) digestible energy (DE; kJ/kg^0.75^/day), (C) crude protein (CP; g/kg^0.75^/day), and (D) digestible protein (DP; g/kg^0.75^/day) from 65 single choice feeding trials conducted in Kluane, Yukon, in February and March of 2022 and 2023. Trend lines, confidence intervals, and *R* ^2^s for linear models are shown. The 0‐line represents 0% weight change.

As expected, the interpretation of our results changed on the basis of whether digestible or crude intake was used. When plotted as GE and CP, intake rates from no‐choice trials showed a concave curve, pointed away from the target intake (Figure 3B), which is not described as a rule of compromise in NG (Simpson and Raubenheimer 2012). This concave curve makes it appear as though hares are fed to meet a minimum protein intake on lower protein diets and fed to meet a minimum energy intake when on higher protein diets. However, this response changed considerably when plotted in digestible terms, showing a non‐interacting effect whereby hares fed to meet a minimum DE intake of approximately 1000 kJ/kg^0.75^/day (Figure 3C), which was very similar to the DE requirement estimated via linear regression (Figure 7B). Hares seemed to attempt to meet this DE requirement on the lower quality diet (Diet A) by consuming more dry matter, but could not consume enough to maintain their weight, likely because the diet had very low dry matter digestibility (Figure 5). Other work on snowshoe hares did not find evidence of compensatory feeding but observed that total consumption rate did not affect weight change, likely for similar reasons (Rodgers and Sinclair 1997; Sinclair et al. 1982).

We found a narrower range of weight maintenance when performance was mapped using DE and DP intakes compared to GE and CP (Figure 6). Specifically, in crude intake rates, the heat map shows excess GE impedes performance relative to protein (Figure 6A), likely because GE includes all sources of carbon, even structural carbohydrates that are poorly digestible, thus are not usable by the hares. This confound is precisely why GE is not regarded as a meaningful food component in traditional terrestrial herbivore studies. When using the more biologically relevant metric of DE, higher energy intake does result in improved performance (Figure 6B). Our results emphasize that for vertebrate herbivores, the effects of fiber on food digestibility should be accounted for when using the geometric framework to assess nutrient targets. For herbivores like hares that consume forbs and evergreen and deciduous shrubs, the capacity to precipitate protein by condensed tannins in plants should also be accounted for because it reduces protein and energy digestibility (DeGabriel et al. 2009) and influences forage palatability (Freschi et al. 2025; Rizzardini et al. 2019; Rossa et al. 2025).

Our estimate for snowshoe hare protein requirement was more realistic to natural foods compared to other studies. We found that hares required a CP composition between 6.5% and 8.6% to maintain weight, which is within the range found in dormant twigs in our study area. Rodgers and Sinclair (1997), who used twigs as food rather than formulated diets, found a higher threshold of 9.9% CP, above that of twigs. Their study used longer feeding trials than ours, so we expect that some variation in our weight change results is due to water weight. Hares cannot subsist on any single species of twig alone, possibly because of plant secondary metabolites in twigs (Bryant and Kuropat 1980; Rodgers and Sinclair 1997). Hares likely maintain a diet diverse in species to not overconsume one type of secondary metabolite (Bernays et al. 1994), explaining the higher protein requirement estimated by Rodgers and Sinclair (1997).

**FIGURE 8:**
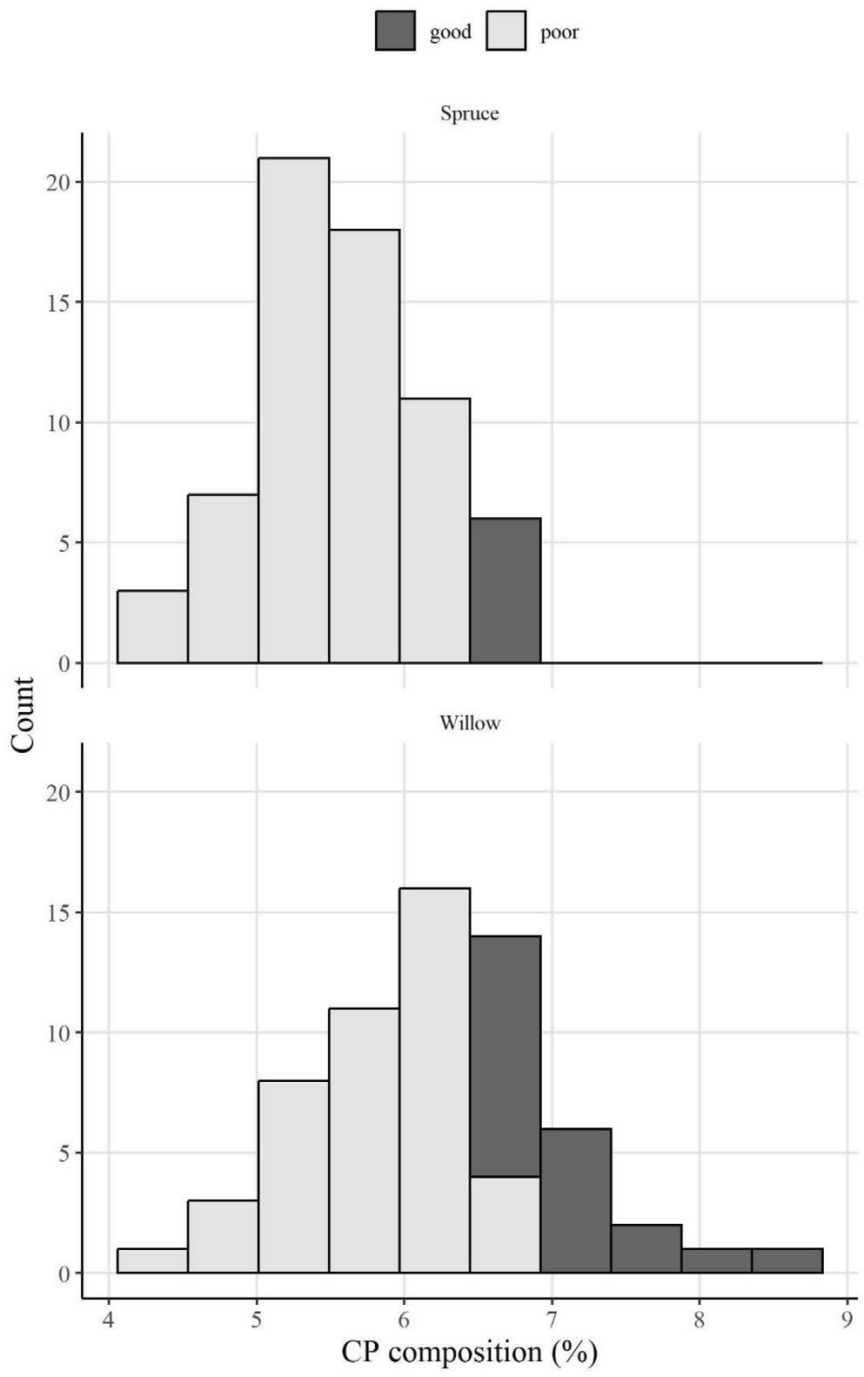
The distribution of crude protein (CP; %) compositions from samples of white spruce trees ( *Picea glauca* ; *n* = 66) and willow shrubs (*Salix* spp.; *n* = 63) in Kluane, Yukon. Twigs (≤ 4 mm) were clipped pre‐snowfall in autumn of 2024 from branches that were less than two meters tall. Bars are shaded according to a quality threshold to exemplify the low availability of sufficient protein content within twigs. Using a conservative estimate, we classified any sample having a protein content greater than that of Diet A from feeding experiments (6.5% CP) as “fair quality” (dark gray) and anything less as “poor quality” (light gray). This classification is based on the feeding trial results of weight change in response to diet, or treatment, which showed that hares lost weight on Diet A, but maintained weight on Diet B. We do not know the threshold between these diet compositions that would result in weight maintenance, which is why we consider this a conservative estimate.

Nutrient limitation could play a role in the snowshoe hare population cycle. Our results support the notion put forth by Sinclair et al. (1982) that hare selection for twig protein likely causes the average twig protein content to decline as the hare population undergoes as much as a 40‐fold change in density (on the basis of optimal foraging theory; Pyke et al. 1977). This decline in food quality at peak densities could contribute to the subsequent increase in over‐winter weight loss that occurs when the population crashes (Sinclair et al. 1988). We have observed that willow twigs vary in protein content, ranging from 4.2% to 8.5% CP (Figure 8). Thus, even willow, one of the lower quality species in our study area, does occasionally produce twigs with higher protein concentrations that hares should strongly select. Food limitation was eliminated as a necessary mechanism for the hare cycle because food supplementation did not affect the timing of the population crash (Krebs et al. 2018). However, supplementing with food did reduce over‐winter weight loss in all experiments and analyses (Hodges et al. 2006; Majchrzak et al. 2022; Sinclair et al. 1988), and food quality, as a limitation, was never thoroughly examined. Future work should assess, in detail, variation in twig quality and how it changes with hare density and other environmental factors such as how snow depth affects availability.

Food supplementation experiments do not just supply animals with more food; they allow animals to meet a target intake more easily than in natural food conditions (Felton et al. 2017). Most supplementation experiments use high‐energy and low‐fiber foods that have different nutritional profiles compared to natural foods (Boutin 1990; Fortier and Tamarin 1998). We designed Diet D to represent rabbit chow used in the previous food supplementation experiments on snowshoe hares (Hodges et al. 2006; Majchrzak et al. 2022; Murray 2002; Shiratsuru et al. 2021). Diet D alone is an unbalanced food for hares; we found that hares must feed more on Diet D than on a diet closer to their target intake to maintain weight (Figure 6). However, having access to food like Diet D, combined with natural foraging, would allow a hare to meet the target intake that is above the protein: energy ratio of dormant twigs. This explains why food supplementation causes animals to forage less, reduce their home range size, increase their body weights, and have advanced breeding compared to control animals (Majchrzak et al. 2022). According to the NG theory, a food supplementation experiment that uses foods closer to the animal's target intake could find a more negative effect of food on space use and foraging rates than these traditional studies (Felton et al. 2017).

Because of the difficulty in conducting captive feeding trials with wild‐caught snowshoe hares, our methods had several limitations. Firstly, our sample size of snowshoe hares was relatively low because our study period corresponded with the low and early increase phase of the snowshoe hare population cycle. In the winter of 2022, our mark‐recapture census estimated only six hares per 100 ha (Krebs et al. 2023). Given this, our sample size of eight hares of adequate body weight for feeding trials likely reflected a high proportion of the available population. Additionally, as in all nutritional studies with captive animals on pelleted diets, we might expect some differences in intake, in vivo digestibility, and energy expenditure. However, previous work has found that the feeding rates and digestibility of twig species tested in feeding trials corresponded to the natural foraging patterns of snowshoe hares (Ellsworth et al. 2013), and this approach allowed us to explore hare physiology and decisions without confounding factors such as predation (e.g., Hodges and Sinclair 2003) and abiotic winter conditions. Further, although weight change has been used as an index of body condition of snowshoe hares in other feeding studies (Rodgers and Sinclair 1997; Sinclair et al. 1982) and has been correlated with hare survival (Hodges et al. 2006), weight changes over a short 3‐day trial may be influenced by short‐term changes in water and food in the digestive system as well as actual changes to protein and fat stores (Barboza et al. 2009). We chose the 3‐day period to reduce the total time in captivity. Lastly, our study design only manipulated ratios of protein and energy and did not account for other food components like minerals (e.g., Villalba and Provenza 2007) or plant secondary metabolites (e.g., Bryant and Kuropat 1980). Although protein and energy have repeatedly been shown to affect hare feeding choices and weight outcomes, future studies could evaluate the influence of other nutritional components that are common in the natural diets of hares. NG offers a means to display how animals respond behaviourally or physiologically to multiple nutrients simultaneously, an otherwise difficult task (Simpson and Raubenheimer 2012). In our case, we used NG to evaluate how snowshoe hare nutrition and body condition were influenced by the interaction of energy and protein, which allowed for a more detailed analysis than possible in other studies (Rodgers and Sinclair 1997; Sinclair et al. 1982). Although NG is a powerful framework, using biologically relevant metrics in nutritional models for vertebrate herbivores remains critical.

## Author Contributions

**Juliana Balluffi‐Fry:** conceptualization (lead), data curation (lead), formal analysis (lead), funding acquisition (lead), investigation (lead), methodology (lead), project administration (lead). **Lisa Shipley:** methodology (equal). **Ruurd T. Zijlstra:** conceptualization (equal), methodology (equal). **Edward W. Bork:** conceptualization (equal), methodology (equal). **Murray Humphries:** conceptualization (equal), methodology (equal). **Stan Boutin:** conceptualization (equal), formal analysis (equal), funding acquisition (equal), investigation (equal), methodology (equal).

## Conflicts of Interest

The authors declare no conflicts of interest.

## Supporting information


**Appendix S1:** ece372347‐sup‐0001‐AppendixS1.docx.

## Data Availability

All data are available at https://zenodo.org/records/17138232.
