# Supplementary material for: Nutrient Balancing by a Wild Browsing Herbivore: Nutritional Geometry of Snowshoe Hares (Lepus americanus)
Source: Ecol Evol. 2025 Oct 18;15(10):e72347. doi: 10.1002/ece3.72347 (PMC12535209; doi:10.1002/ece3.72347)
Supplement: Supplementary file 1 — Appendix S1: ece372347‐sup‐0001‐AppendixS1.docx. [file ECE3-15-e72347-s001.docx]

Supplementary information


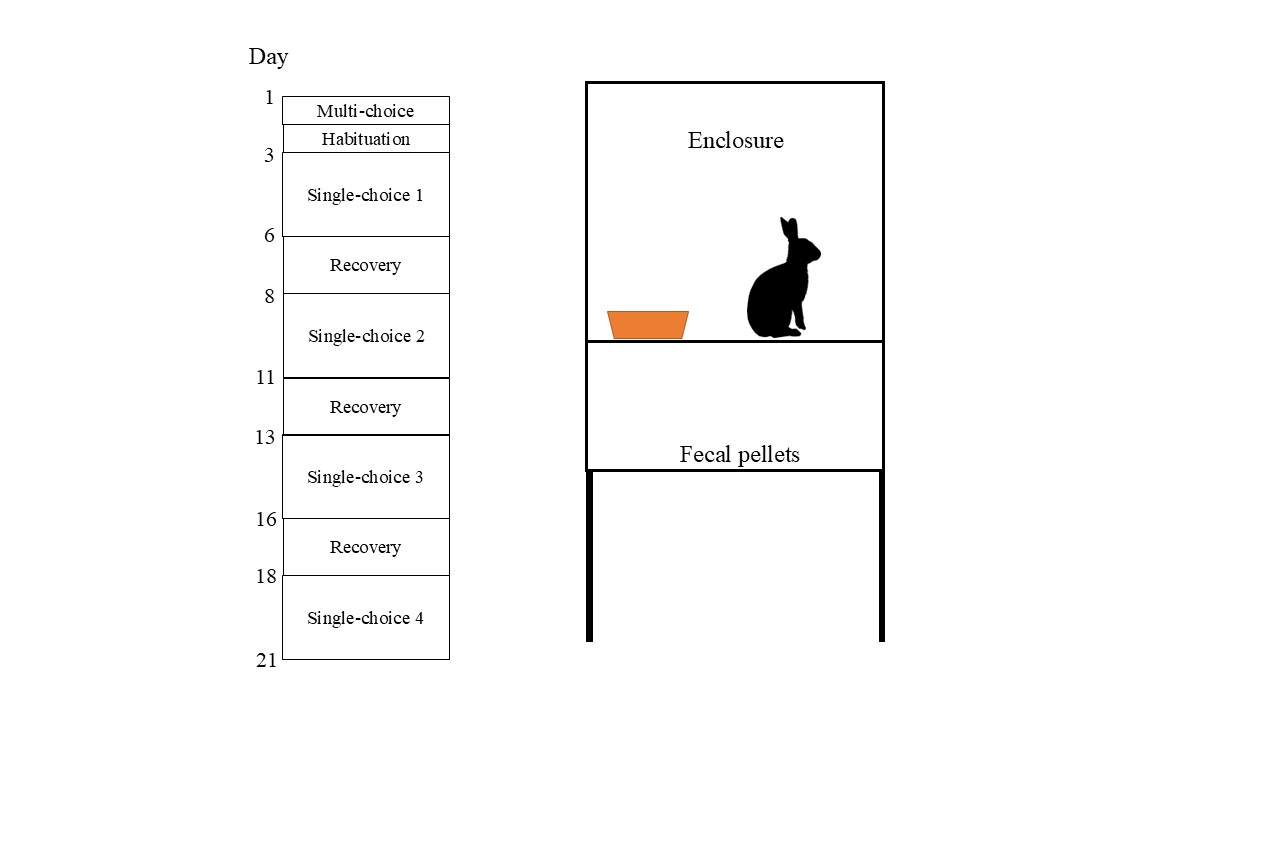


Figure S1. Experimental procedure (left) and enclosure design (right) for snowshoe hare feeding trials. Experimental procedure is blocked by phase boxes, which are scaled to their length in days. On each labeled day, hare weight was measured.
